# FlexPlex27—highly multiplexed rapid DNA identification for law enforcement, kinship, and military applications

**DOI:** 10.1007/s00414-017-1567-9

**Published:** 2017-03-03

**Authors:** Ranjana Grover, Hua Jiang, Rosemary S. Turingan, Julie L. French, Eugene Tan, Richard F Selden

**Affiliations:** ANDE, 266 Second Avenue, Waltham, MA 02451 USA

**Keywords:** Rapid DNA identification, CODIS 20, Kinship, Short tandem repeat

## Abstract

Rapid DNA identification is the use of a rugged, field-deployable system to generate short tandem repeat (STR) profiles in law enforcement, military, immigration, and homeland security applications. A performance verification study was conducted on the ANDE Rapid DNA identification system using FlexPlex27, a highly multiplexed, 27 locus assay that generates data for the expanded CODIS core loci and all additional STR loci required for international databasing. The assay contains 23 autosomal loci (D1S1656, D2S1338, D2S441, D3S1358, D5S81, D6S1043, D7S820, D8S1179, D10S1248, D12S391, D13S317, D16S539, D18S51, D19S433, D21S11, D22S1045, FGA, CSF1PO, Penta E, TH01, vWA, TPOX, and SE33), three Y-chromosomal loci (DYS391, DYS576, and DYS570), and Amelogenin. Study results demonstrate that the instrument is reliable, reproducible, accurate, robust, and ready for a large scale, comprehensive developmental validation by NDIS-participating laboratories. The additional loci in the FlexPlex assay allow for improved STR profile sharing globally, increase the power of discrimination for identification matches, and improve the effectiveness of kinship analyses.

**Electronic supplementary material:**

The online version of this article (doi:10.1007/s00414-017-1567-9) contains supplementary material, which is available to authorized users.

## Introduction

Short tandem repeat (STR) analysis is applied in a wide range of disciplines, including law enforcement, paternity testing, disaster victim identification, and the military. Based on a series of advances over the past two decades, STR analysis in the laboratory is now well-established, and it is not surprising that the utility of STRs is broadening beyond the laboratory. Perhaps two extensions of the reach of STR analysis are most important in this regard: Rapid DNA identification and expanded sets of core STR loci.

Rapid DNA identification can be defined as the fully automated generation and interpretation of STR profiles (colloquially termed “DNA fingerprints”) in less than 2 h in a ruggedized, field-deployable system by nontechnical users. The potential impact of rapid DNA technology is evidenced by the fact that the Department of Defense, the Federal Bureau of Investigation (FBI), and the Department of Homeland Security have collaborated to develop a series of requirements for rapid DNA Identification systems [1]. Furthermore, the FBI’s establishment of the Rapid DNA Index System [2] (RDIS), the unanimous passage of the Rapid DNA legislation in the Senate [3] and in the House Judiciary Committee [4], and the National DNA Index System (NDIS) approval of the ANDE Rapid DNA System (consisting of the fully integrated instrument, consumable microfluidic chip, and Expert System software) [5] suggest that STR generation outside the laboratory may eventually become routine.

In parallel with these developments, several countries have modified and expanded their sets of core STR loci used for databasing and matching against casework, military, and other samples. For example, the FBI has announced the expansion of the US Combined DNA Index System (CODIS) core loci from 13 to 20, with a goal of reducing the likelihood of adventitious matches [6] and increase compatibility of international databases (many of which use distinct sets of STR loci, hindering the comparison and matching of STR profiles across borders). Another major benefit of increasing the number of STR loci is to improve kinship analysis [7, 8] in the identification of missing persons, victim identification, and reunification of family members following mass disasters, processing of refugees and asylees, and immigration applications [9, 10].

Kinship analysis using STRs is based on classical Mendelian genetics. The closer the biological relationship between two individuals, the more alleles they are likely to share. First-degree relatives are those that share approximately 50% of their genetic material—individuals that have parent-offspring and sibling relationships. Paternity testing is the most common kinship application of STR technology. An offspring inherits two alleles at each STR locus, one from each parent—these are “obligate alleles” in the sense that the genome of the offspring must contain these alleles (the exceptions to this rule include small mutations and chromosomal abnormalities). Because of obligate alleles, it is generally straightforward to establish parental-child relationships with an assay which targets the CODIS core 13 loci. In contrast, Butler and colleagues [11] showed that an assay which targets only the CODIS core 13 loci leads to a 2.7% false positive rate and 3.3% false negative rate for sibling pairs; siblings need not inherit the same allele from a given parent. The study also included evaluation of kinship analysis simulations of half siblings; the false positive and negative rates observed in 1000 simulations with the CODIS core 13 STR loci were 15.5 and 17.3%, respectively.

**Fig. 1:**
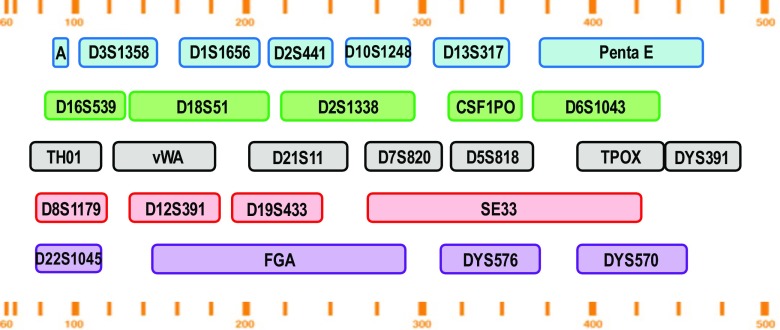
Layout of the STR markers included in the FlexPlex27 assay. Approximate locus ranges are indicated in bases above and below the 27 loci. The assay contains 23 autosomal loci, three Y-chromosomal loci, and Amelogenin

We have developed a multiplex assay which interrogates 27 loci, termed FlexPlex27 (Fig. 1) and adapted it for Rapid DNA Identification in the ANDE 6C System. The assay contains 23 autosomal loci (D1S1656, D2S1338, D2S441, D3S1358, D5S81, D6S1043, D7S820, D8S1179, D10S1248, D12S391, D13S317, D16S539, D18S51, D19S433, D21S11, D22S1045, FGA, CSF1PO, Penta E, TH01, vWA, TPOX, and SE33), three Y-chromosomal loci (DYS391, DYS576, and DYS570), and Amelogenin. In addition to the STR loci of the FBI’s expanded CODIS core loci, FlexPlex27 generates data compatible with the ENFSI/EDNAP Expanded European Standard set and a wide range of national DNA databases including Australia’s National Criminal Investigation DNA Database, Canada’s National DNA Data Bank, China’s National DNA Database, Germany’s DNA-Analyze-Datei, New Zealand’s National DNA profile databank, and the United Kingdom’s National DNA Database.

The initial ANDE instrument (also known as ANDE 4C) performs laser-based detection of four fluorescent dyes and is compatible with the PowerPlex 16 System (Promega Corporation) [12]. To accommodate FlexPlex, the ANDE 6C instrument was designed to permit detection of six fluorescent dyes. Accordingly, the goals of this internal study of the ANDE 6C system and FlexPlex assay were twofold. First, the study was designed to assess the performance of the system in processing buccal swabs in preparation for developmental validation by independent NDIS-participating laboratories and subsequent submission for NDIS approval. For the sake of clarity, the developer-conducted evaluation detailed in this paper will be referred to as a performance verification study (as opposed to validation) to differentiate from the developmental validation conducted by independent NDIS-participating laboratories. Second, the study evaluated the impact of a larger number of STR loci on kinship determination for a range of claimed relationships.

## Materials and methods

### Rapid DNA identification

When operating the ANDE System, five buccal swabs were loaded into each chip which was then inserted into the instrument, and all steps were completed by following onscreen instructions. The instrument automatically processes the swabs to purify the DNA, amplify the purified DNA to generate labeled STR fragments, and separate and detect the STR fragments. The on-board ANDE Expert System automatically performed allele calling and profile interpretation. The total processing time is approximately 90 min per run.

### Buccal swab collection

Buccal swab samples were collected using ANDE swabs as follows. Donors were instructed to press the swab gently against the inside of one cheek, move it in an up-and-down motion six times, and repeat with the other cheek (6 swipe protocol). The swab head is rotated during the collection to ensure that buccal cells are collected around the entire surface area. Other swab types can be used in the system; the advantage of the ANDE swab is that it contains a 2-D barcode and RFID chip in the swab cap that simplifies sample tracking. For concordance purposes, samples processed on the ANDE instrument were also subjected to conventional laboratory testing. The study was approved by an Institutional Review Board, and informed consent was obtained from all individual participants included in the study.

### Reproducibility

Three buccal swabs were collected from each of ten unique donors and processed on three unique ANDE 6C instruments. Concordance, signal strengths, and heterozygote peak height ratios of the three replicate profiles from each donor were compared.

### Sensitivity

Buccal swabs were collected in triplicate from five unique individuals using a 1 swipe, 3 swipe, and 6 swipe protocol. Each set of swabs was processed on a unique ANDE 6C instrument. The resulting profiles from the 15 replicates of each swabbing protocol were assessed for signal strength and heterozygote peak height ratios.

### Species specificity

Duplicate buccal swabs were spiked with DNA from each of ten unique species and processed on ANDE 6C. Genomic DNA from each of ten species was prepared as a 50-μl solution of TE^−4^ and spiked on swabs. The quantity of DNA per species spiked on each swab is as follows. Mouse (*Mus musculus*), Ferret (*Mustela putorius furo*), Horse (*Equus ferus caballus*), and Dog (*Canis lupus familiaris*): 1000 ng of genomic DNA. Orangutan (*Pongo pygmaeus*), Gorilla (*Gorilla gorilla*), and Chimpanzee (*Pan troglodytes*): 500 ng of genomic DNA. *Lactobacillus plantarum*, *Staphylococcus aureus*, and *Streptococcus pneumoniae:* 100 ng of genomic DNA.

### Mixtures

Purified DNA from a male and a female donor was quantified using a Nanodrop 2000C Spectrophotometer (Thermo Fisher Scientific). DNAs from each donor were mixed in ratios of 19:1, 5:1, 1:1, 1:5, and 1:19 yielding a total of 1 μg of DNA in 50 μl of TE-4. Each mixture was pipetted onto an ANDE swab and processed in duplicate. Each profile was reviewed manually and all alleles assigned to either the major or minor donor.

### Inhibitors

Two buccal samples from each individual were collected in the presence of ten unique potentially inhibitory substances: mint, gum, toothpaste, mouthwash, blood, beer, tea, tobacco dip, cigarette, and coffee. The potential inhibitors were consumed or used by the donor immediately prior to buccal swab collection. For example, gum was chewed for approximately 5 min just before standard buccal swab collection was performed. The only exception was that 10 μl of blood from the same donor as the buccal sample was pipetted directly onto the buccal swab following collection and prior to the run.

### Stability

Fourteen buccal swab samples were collected from each of two unique donors. Immediately after buccal swab collection, one set of swabs was stored in a protective clear plastic tube containing desiccant and the other set was stored in the standard protective clear plastic tube (not containing desiccant). For each set of 14 samples, two samples from each donor were processed immediately (fresh), after 1 day of storage at 22 °C, 1 day of storage at 4 °C, 2 days of storage at 22 °C, 2 days of storage at 4 °C, 7 days of storage at 22 °C, and 7 days of storage at 4 °C.

### Contamination

Runs were made with the following three sample loading configurations: blank/blank/blank/blank/blank, sample/blank/sample/blank/sample, and blank/sample/blank/sample/blank. Each loading configuration was performed in duplicate. Buccal swab samples were collected following the standard protocols, and blank swabs were new swabs removed from the packaging and placed directly into the chip.

### First pass success, concordance, and accuracy

Two hundred twenty unique donor samples (44 chip runs) were processed to determine first pass success, concordance, signal strength, and peak height ratio. The first pass success rate was determined by evaluating the number of samples with all CODIS core 20 loci or all FlexPlex27 loci passing on the first run (including fully integrated Expert System interpretation). Concordance was determined by comparing the allele calls generated by ANDE 6C with allele calls of the same donor generated by an outside laboratory using conventional methods.

### Precision and resolution

Precision and resolution were analyzed based on 76 runs (380 samples). Inter-run precision was calculated by determining the standard deviation of the fragment sizes (in bases) of the allelic ladder fragments. Resolution was calculated as previously described [13], with R (resolution) values ≥0.2 indicating single base pair resolution.

### A-Chip

All Rapid DNA Identification was performed within a previously described [12] chip, a single use, disposable consumable that is fabricated by injection molding using cyclic olefin polymer. The chip includes all reagents, microfluidic components, and waste containment required to perform STR analysis. DNA purification reagents, STR reagents, buffers, and separation polymer are all pre-loaded into the chip, and all reagents are stable for at least 6 months at room temperature [12]. Referred to as the “A-Chip,” the FlexPlex chip is structurally identical to the PowerPlex 16 chip that has received NDIS approval [5]; the only differences are the incorporation of lyophilized reagents for the FlexPlex27 PCR assay and the WEN Internal Lane Standard (ILS) 500 (Promega Corporation).

### Instrument

The ANDE 6C instrument is based on the previously described ANDE 4C [12] instrument. The major enhancements in the new system are (1) the ability to perform STR analysis with either four or six fluorescent dye labels, enabling the generation of STR profiles with the expanded CODIS core loci, (2) the addition of a 2-D barcode scanner for sample tracking, and (3) enhanced ruggedization for utilization outside the laboratory and for mobile applications. The new instrument was designed to minimize changes to the core design of the ANDE 4C system. There were no changes made to the mechanical interface between the instrument and chip, pneumatic subsystem, thermal cycling subsystem, and high voltage subsystems. The major enhancements in the ANDE 6C optical system are the incorporation of two additional detector modules and dichroic mirrors (for a total of six each). These elements are configured to detect two additional dye colors (purple and orange) for a total of six colors. The detectors and dichroic mirrors cutoff wavelengths and pathlengths for the blue, green, yellow, and red dyes are identical in the ANDE 4C and ANDE 6C systems.

Note that all ANDE chips, whether for buccal swab samples [12], casework, military, disaster victim identification, or other non-buccal samples [14], and using either the PowerPlex 16 assay or the FlexPlex assay, maintain the identical chip-to-instrument interface and can be processed using the same ANDE 6C instrument. The instrument detects the chip type using an internal RFID reader and automatically selects the required sample processing protocol. This paper reports on the performance of the buccal swab FlexPlex chip (A-Chip).

### Expert system

The ANDE 6C Expert System is based on the NDIS-approved 4C Expert System [5, 15], with the only differences being the added capabilities of processing the two additional dye colors and interpreting the three hemizygous loci. The software processes the raw data, assigns allele designations, and employs rules to interpret the DNA profiles. The Expert System software was specifically designed and developed for the analysis of ANDE data and is fully integrated with no user intervention required. Immediately following an ANDE run, optical data generated during electrophoresis is subjected to signal processing, which includes setting the baseline to zero and performing color correction. The Expert System then evaluates the internal lane standard and the allelic ladder using a strict set of criteria. Then, a series of rules are applied to assign alleles and evaluate locus- and sample-specific criteria such as peak height, stutter, and heterozygote peak height ratio. At the conclusion of the evaluation, the ANDE Expert System generates the following outputs:

- An allele table listing all passing allele calls for all samples
- A .png file (electropherogram) for rapid output visualization
- An .xml file for upload to Combined DNA Index System (CODIS)
- An .fsa file to permit review with conventional software packages

### Conventional laboratory testing

One swab collected from each unique donor from the following studies was sent for conventional laboratory STR testing: reproducibility, sensitivity, stability, mixtures, inhibitors, stability, contamination, and accuracy. DNA was extracted from the buccal swabs using the BioSprint 96 Robotic Workstation (Qiagen). Approximately 0.5 to 2.0 ng of the extracted DNA was amplified using the PowerPlex Fusion 6C and PowerPlex 21 Systems (Promega) and detected using an Applied Biosystems® 3500 Genetic Analyzer (Life Technologies). The data was analyzed using Genemapper® ID-X, version 1.1 (Life Technologies), and interpreted by two qualified analysts. Additional validated techniques such as reinjection, reextraction, quantification, and increased cycle numbers were employed for samples that did not initially pass technical specifications for reporting.

### Data management in local and central databases

Profile storage and management was performed with ANDE Data Management Software (ADMS). Data generated on multiple ANDE systems was transferred to ADMS through a local area network. The ADMS stores profile data in an onboard database and allows individual profiles to be viewed, exported, and deleted. The software also allows DNA databases to be created, imported, exported, and deleted.

Comparisons between profiles may be made by exporting to a central database or locally using ADMS, which allows automatic searching and matching of newly imported profiles against profiles that are stored in database. The match between a new profile and a profile in the database is determined by first calculating the following at each locus:

- Number of loci available for matching. A locus is defined as available for matching when it has allele calls in both the sample profile and the database profile.
- Number of loci that match. A locus is classified as a match when both alleles of the sample profile and the database profile are identical.
- Number of loci with drop-ins or drop-outs. A locus is classified as a drop-in or drop-out when one of the alleles matches, but other allele does not match.
- Number of mismatched loci. A locus is classified as a mismatch when both alleles of the locus do not match.

A final result of match, possible match, or mismatch is assigned based on the match criteria as defined by the jurisdiction.

### Kinship analysis

Kinship analysis was performed implementing eDNA software (Genetic Technologies, Inc.). The kinship module has been validated to calculate likelihood ratio, paternity index-PI, and combined relative index-CRI, and STR profiles from two individuals when compared to calculate the likelihood of the main hypothesis (i.e., the two individuals have a family relation) versus the likelihood of alternate hypothesis (i.e., the two individuals are unrelated). eDNA software uses established kinship formulas [16, 17] and statistical analysis to calculate likelihood ratios for a wide range of relationships. The formulas used to calculate likelihood ratio are dependent on the given STR genotypes of two individuals, respective allele frequencies, and the probabilities that 2, 1, or 0 alleles have shared identity by descent given a specified relationship. Assuming 50% prior probability, the posterior probability of the main hypothesis (W) is derived from this likelihood ratio-LR or PI or CRI by dividing LR/(LR + 1). Allele frequencies used for kinship analysis were based on Promega allele frequencies [18–20]. Kinship analysis was performed for relationships including full siblings (50 pairs), half siblings (7 pairs), and grand-parent/grand-child (22 pairs). Calculations were performed with FlexPlex27, CODIS core 13, and CODIS core 20. Due to the linkage disequilibrium between VWA and D12S391 [21] and between D6S1043 and SE33 [22], D12S391 and D6S1043 were excluded from FlexPlex27 and CODIS core 20 kinship calculations.

## Results and discussion

### Reproducibility

**Fig. 2:**
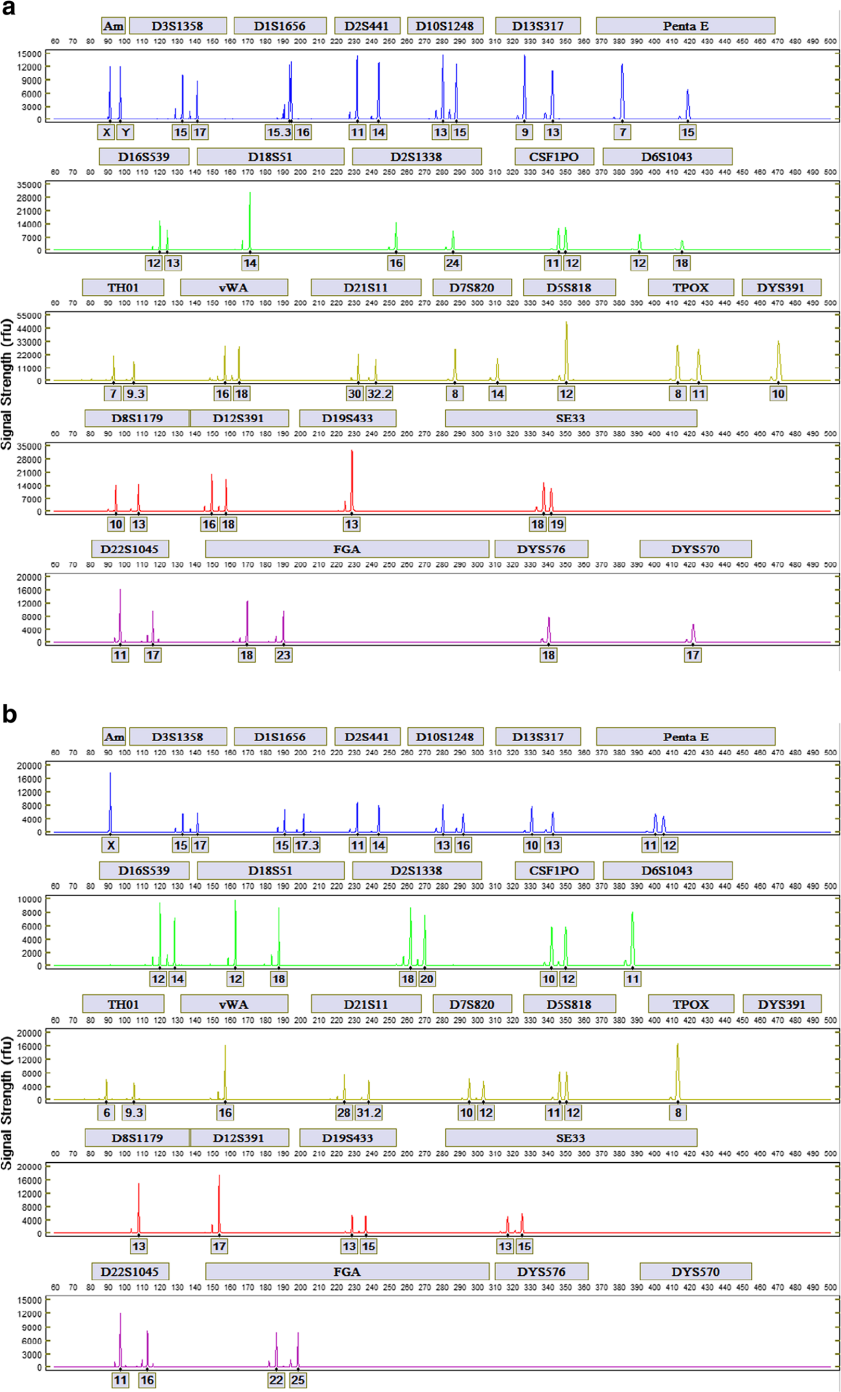
STR profiles for **a** a male donor and **b** female donor generated with the FlexPlex27 assay and ANDE 6C instrument

**Fig. 3:**
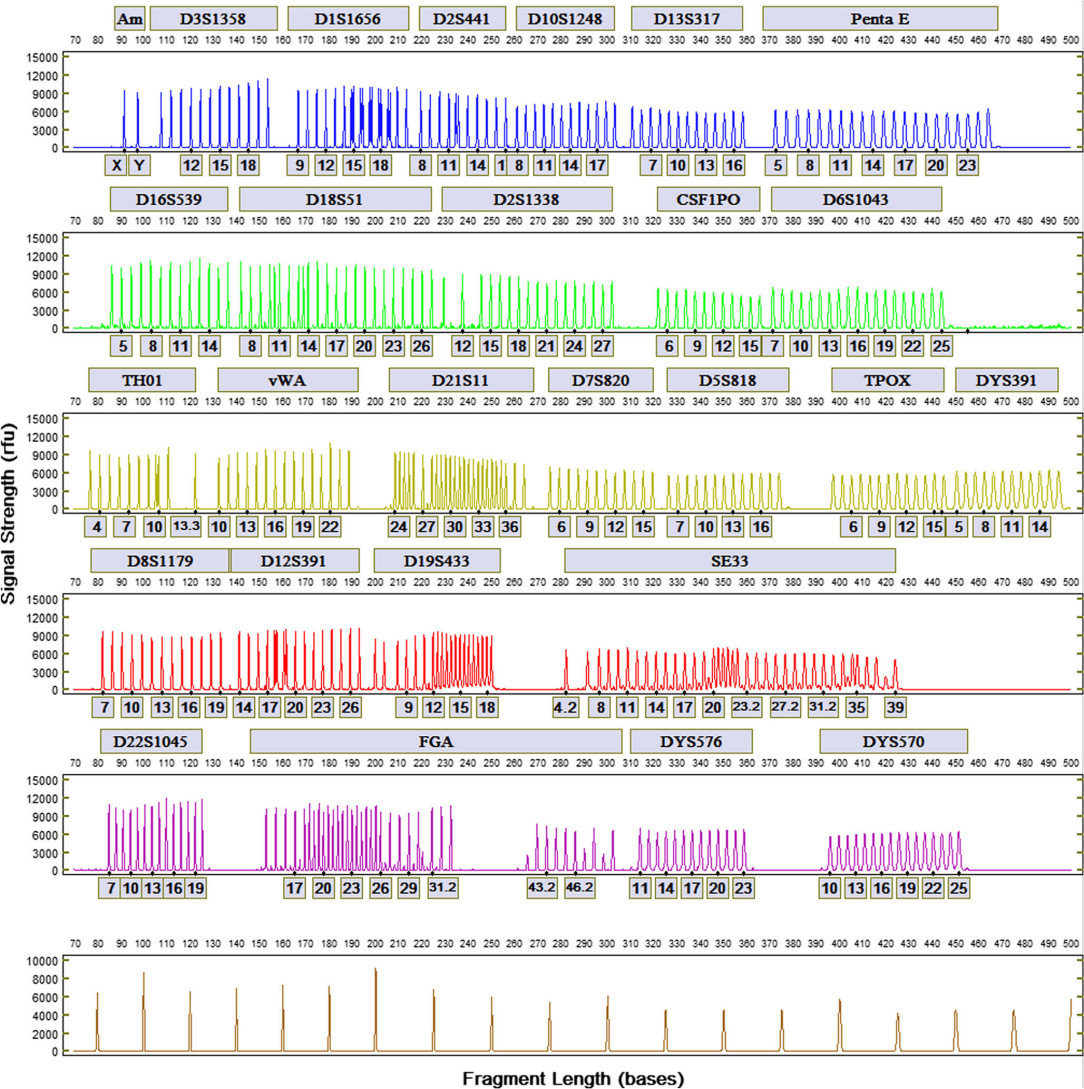
Allelic ladder for the FlexPlex27 assay

**Fig. 4:**
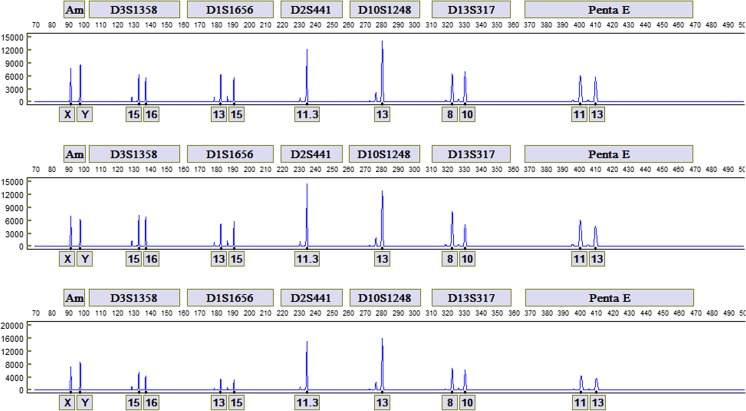
Comparison of the profiles from the blue dye channel for one buccal donor on three different ANDE 6C instruments

STR profiles from a male and female donor generated with the FlexPlex27 assay are shown in Fig. 2a, b, respectively, and the Allelic Ladder is shown in Fig. 3. Reproducibility was demonstrated by the analysis of STR profiles with the expected genotypes obtained in triplicate from 10 donors. For each donor, profiles generated from three different ANDE 6C instruments were concordant with each other and with conventional laboratory results. The rapid DNA profiles from the blue dye channel for one buccal donor using three ANDE 6C instruments are shown in Fig. 4. Signal strengths and heterozygote peak height ratio were comparable across the three instruments for each donor sample.

### Sensitivity

**Fig. 5:**
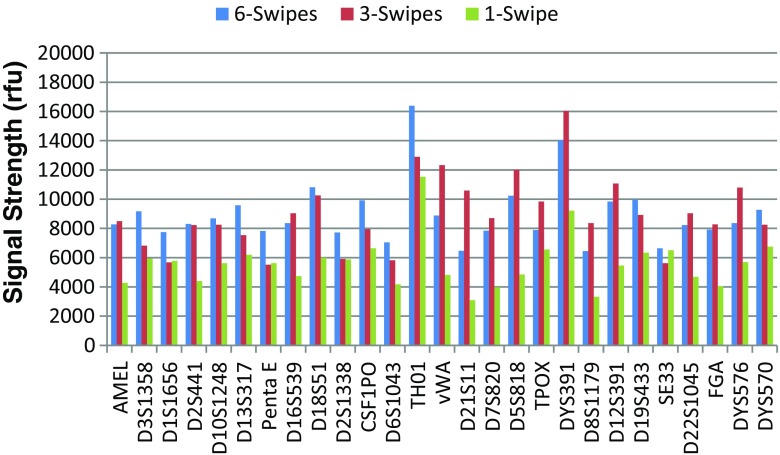
Comparison of signal strengths for buccal samples collected using six swipe, three swipe, and one swipe protocols

Sensitivity of the system was assessed studying the impact of 1, 3, and 6 swipes collection protocol on signal strength and peak height ratio. Full CODIS core 20 profiles were generated for all 45 samples processed on three different ANDE 6C instruments, and all profiles were concordant with those generated by conventional testing. There was no apparent difference in the signal strengths between samples collected with a 6 swipe and 3 swipe protocol, and there was a decrease in signal strength for samples collected with a 1 swipe protocol (Fig. 5). No difference in heterozygote peak height ratios were observed across the three collection protocols. Taken together, the data shows that the system is relatively insensitive to the number of swipes (6 swipes, 3 swipes, or 1 swipe) used to collect buccal swab samples.

### Species specificity

Specificity of the FlexPlex assay to human samples is essential to ensure that interpretation of STR profiles derived from human subjects is not complicated by cross-reactivity from non-human samples. A set of 10 relevant organisms including bacteria, domestic animals, and primates were evaluated in duplicate, and none yielded a passing profile. Profiles generated for Mouse (*Mus musculus*), Ferret (*Mustela putorius furo*), Horse (*Equus ferus caballus*), Dog (*Canis lupus familiaris*), *Lactobacillus plantarum*, *Staphylococcus aureus* DNA, and *Streptococcus pneumoniae* did not contain any called peaks. Profiles generated from primates Orangutan (*Pongo pygmaeus*), Gorilla (*Gorilla gorilla*), and Chimpanzee (*Pan troglodytes*), which possess genetic similarities with humans, have no called alleles, but a significant number of alleles labeled in red warning boxes. Alleles in red warning boxes are not considered as passing by the Expert System and are not included in the database-compatible output file.

### Mixtures and inhibitors

The buccal ANDE 6C Expert System was designed for single source samples and is programmed to identify and fail mixed profiles. For the ten mixture samples processed, the expert system reliably detected and failed mixtures when at least two loci had three alleles or one locus had four alleles. All called alleles were those of the major contributor and were concordant with conventional laboratory results. None of the unique alleles from the minor contributor were called. All 20 samples with potential inhibitors generated full, concordant profiles.

### Stability and contamination

The stability of buccal samples collected and stored using ANDE swabs with and without desiccant at 22 and 4 °C for 1, 2, and 7 days was assessed in comparison to the buccal swabs processed immediately after collection. All samples stored at different temperatures and days generated full, concordant profiles similar to those processed immediately after collection. None of the profiles showed effects of degradation from storage.

Sample contamination between runs and between lanes within a run was assessed. The criteria for a successful blank samples were the success of the ILS, the absence of called peaks, and the absence of peaks labeled in red warning boxes. No run-to-run or lane-to-lane contamination was observed.

### Signal strength and peak height ratio

**Fig. 6:**
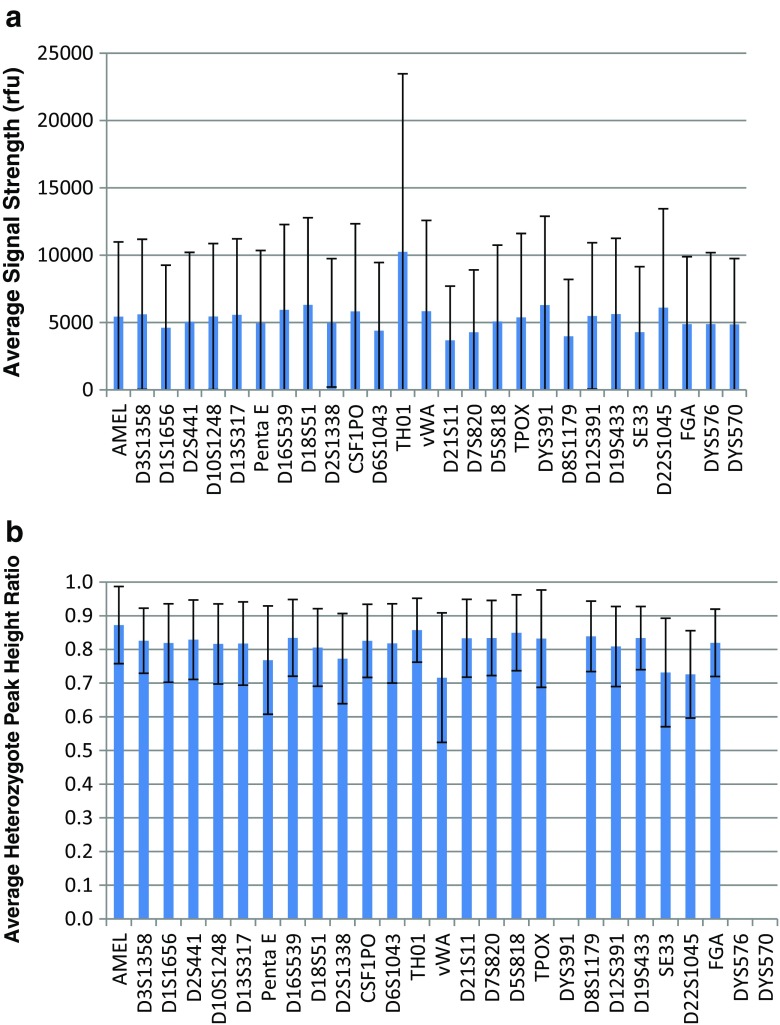
**a** Average signal strength from 220 STR profiles processed with the FlexPlex27 assay and ANDE 6C Rapid DNA system. Standard deviations are indicated. **b** Average heterozygote peak height ratio from the same set of STR profiles as in Fig. 5a. Standard deviations are indicated

The signal strength for each locus was calculated for each of the 220 donor samples by summing the signal strengths of all called peaks within the locus and dividing by two. The average peak height ranged from 3675 rfu at D21S11 to 10,249 rfu at TH01 (Fig. 6a). The heterozygote peak height ratio for each locus was calculated for each of the 220 donor samples by dividing the height of the smaller peak by the height of the larger peak. The average peak height ratio ranged from 0.716 at vWA to 0.857 at TH01 (Fig. 6b).

### Precision and resolution

**Fig. 7:**
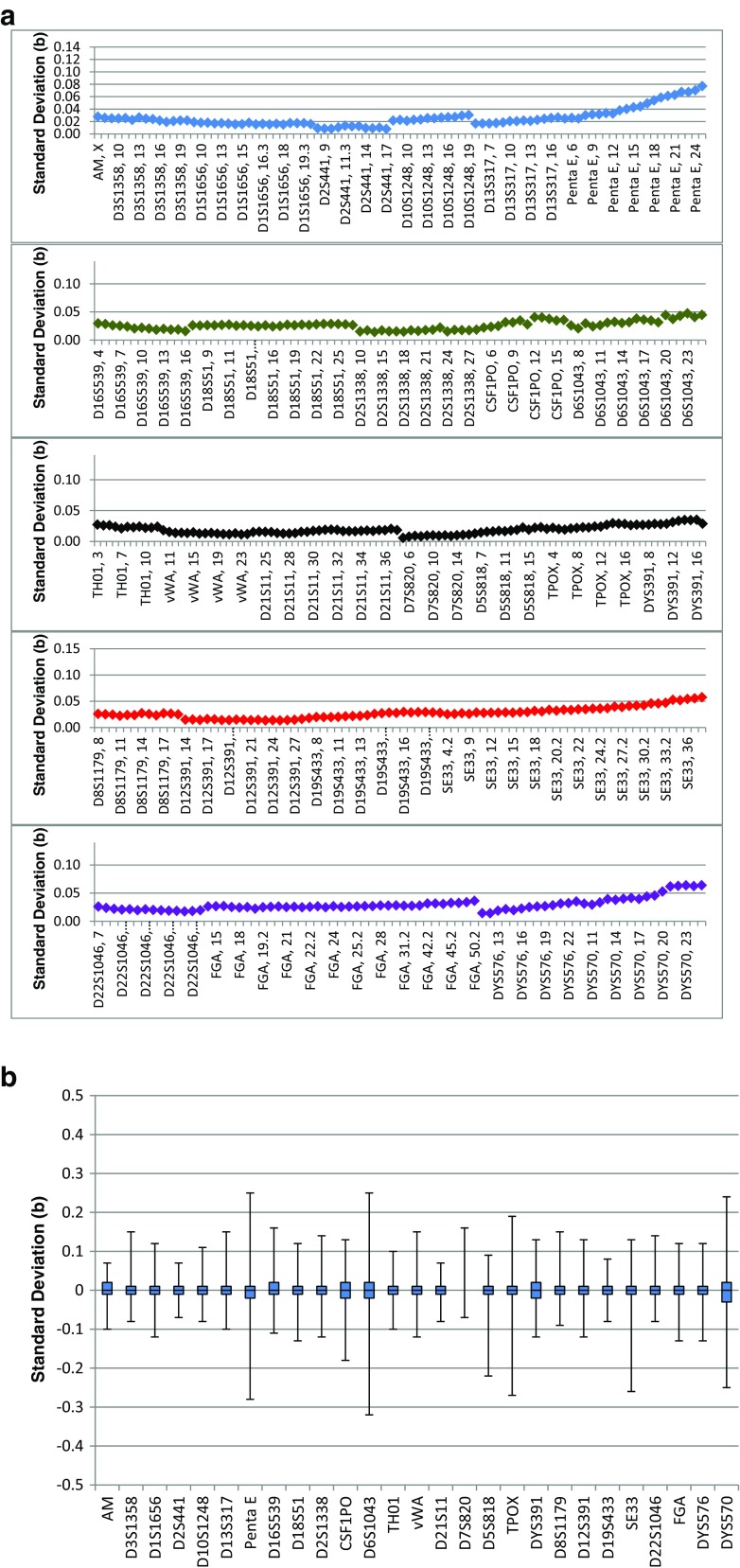
**a** Inter-run precision calculated from 3 ANDE instruments. Standard deviations are indicated. **b** Inter-run precision calculated from three ANDE instruments. Box plot of fragment size deviation of all loci within a locus show that none of the alleles deviate by more than 0.5b

Inter-run precision was calculated for all three instruments used in this study and was determined based on allelic ladders from 76 runs (Fig. 7). The standard deviation in bases was calculated for each allele in the allelic ladder and ranged from 0.006 bases at D7S820 to 0.077 bases at Penta E. The variation at three standard deviations ranges from 0.017 bases to 0.231 bases and are well below the acceptable target value of 0.5 bases.

**Fig. 8:**
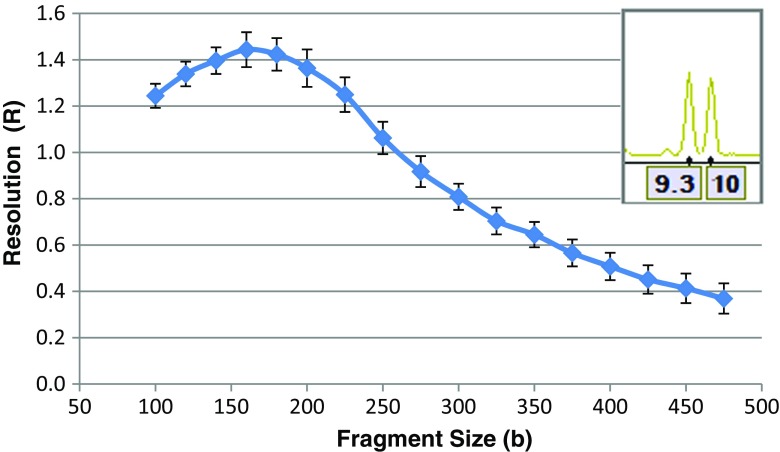
Resolution calculated from 380 samples. *Inset* shows single base pair resolution at the TH01 locus

Resolution calculated based on the 380 samples shows that the system is capable of resolving single base pair differences in fragments across the entire separation range to greater than 500 bases (Fig. 8). The system’s ability to resolve a single base pair difference, such as the 9.3 and 10 alleles at TH01, is also shown (Fig. 8, inset).

### First pass success and concordance

The first pass success rate, defined as the frequency that the first ANDE run generates full STR profiles, was 92.73 and 90.46% for the CODIS core 20 loci and the FlexPlex 27 loci, respectively. The first pass success rate for ANDE 6C is higher than that of the conventional laboratory, which was 81.27%.

A total of 204 samples were assessed for concordance of the CODIS 20 loci, and 199 samples were assessed for concordance of the FlexPlex 27 loci. All loci from all samples were concordant with the exception of an allele dropout at Penta E in a single sample (Table 1). The FlexPlex alleles as determined by the ANDE 6C Rapid DNA Identification system show greater than 99.99% concordance with conventional laboratory analysis.

**Table 1:** Comparison of concordant and discordant alleles observed with CODIS 20 core loci and FlexPlex 27 loci

	CODIS 20 core loci		FlexPlex 27 loci	
	Number of samples	Percentage of samples	Number of samples	Percentage of samples
Samples assessed for concordance	204		199	
Concordant samples	204		198	
Concordant loci	4080		5345	
Concordant alleles	8160		11,342	
Discordant loci	0	0.000%	1	0.019%
Discordant alleles	0	0.000%	1	0.009%

The FlexPlex alleles as determined by the ANDE 6C Rapid DNA Identification system show greater than 99.99% concordance with conventional laboratory analysis

### Kinship analysis

Kinship analysis was performed utilizing three sets of STR loci: CODIS 13, CODIS 20, and FlexPlex27. Supplementary Table 1 shows that of 50 sib-pairs studied, 38 had kinship probabilities of greater than 99.5% using CODIS 13, 44 using CODIS 20, and 46 using FlexPlex. Furthermore, even in the cases for which the kinship probabilities exceed 95%, likelihood ratios attained with FlexPlex are generally much higher than for CODIS 13 and CODIS 20. There were only four sib-pairs for which the kinship probability with FlexPlex was less than 99.5%. These four sib-pairs had kinship probabilities of 11.21, 53.75, 74.28, and 98.94%.

Supplementary Table 2 shows that of seven half-sibling pairs, 1 had a kinship probability of greater than 99.5% using CODIS 13, 2 using CODIS 20, and 3 using FlexPlex. In three other cases, the kinship probabilities attained with FlexPlex was less than 99.5% but greater than both CODIS 13 and CODIS 20 probabilities. In one case, the kinship probability observed with CODIS 13 and CODIS 20 was higher than FlexPlex; however, all three were in the 90–95% range. As observed with true sib pairs, half-sib pairs also showed an order of magnitude or greater likelihood ratios with FlexPlex as compared with CODIS 13 and CODIS 20, thereby, increasing the level of confidence in proving the relationship in question between the two individuals.

Supplementary Table 3 shows that of 22 grand-parent/grand-child pairs, 3 had a kinship probability of greater than 99.5% using CODIS 13, 4 using CODIS 20, and 8 using FlexPlex. In six other cases, the kinship probabilities attained with FlexPlex are less than 99.5% but greater than both CODIS 13 and CODIS 20 probabilities. Once again, the likelihood ratios calculated with FlexPlex are much greater than those achieved with either CODIS 13 or CODIS 20. There is one pair for which the CODIS 13 marker set performs slightly better than FlexPlex (99.75 vs 99.18%).

## Conclusions

The FlexPlex assay has been designed to enable Rapid DNA Identification while incorporating the STR loci of the FBI’s expanded CODIS core set and all other major international STR sets including Australia, Canada, China, ENFSI/EDNAP, Germany, New Zealand, and the UK. The data presented here demonstrates that the FlexPlex assay, in tandem with the ANDE 6C instrument, Expert System, and A-Chip, generates STR profiles in accordance with the SWGDAM Validation Guidelines for reproducibility, sensitivity, accuracy, concordance, precision, resolution, peak height ratio, species specificity, mixtures, inhibitors, stability, and contamination.

The ANDE 6C Expert System reliably processes raw data, assigns allele designations, and employs rules to interpret the resulting profiles. This automated analysis leads to accurate and concordant profiles without the need for interpretation by forensic analysts, a critical requirement for Rapid DNA Identification in police stations, the battlefield, borders and ports, immigration offices, and other field-forward settings. Taken together, the data provides the support for the initiation of a developmental validation study of the system using independent governmental forensic laboratories and subsequent submission to NDIS.

The movement of individuals and families around the globe continues to be a major issue in international relations and humanitarian efforts. Regional political and economic instability, both with and without armed conflict, has contributed to increased numbers of refugees and unaccompanied minors crossing borders, which in turn places significant pressure on governmental authorities to process and identify the large numbers of people entering a given country. In certain instances, the determination of kinship is a critical step in immigration. For example, the US Department of State utilized STR analysis in various kinship applications and requires proof of biological relationship through DNA testing a 99.5% minimum probability of paternity/maternity relationships [23]. Recently, the Bureau of Immigration Appeals found [24] that a 99.5% or greater probability should be accepted for claimed sibling relationships. In this study, we have shown that the use of the FlexPlex assay is substantially more effective at resolving cases subjected to this probability and, accordingly, that rapid DNA may play a significant role in improving the efficiency of immigration processing.

Based in part on the data presented herein, the ANDE 6C/FlexPlex System is undergoing developmental validation by a team of accredited government forensic laboratories, including NDIS-participating laboratories in the USA as a step towards NDIS approval. If the Rapid DNA Act before the US Congress is enacted and signed by the President, routine Rapid DNA Identification at police booking stations and access to the RDIS component of CODIS for real-time searching of the national database would be allowed. In other settings such as disaster victim identification and casework analysis, FlexPlex STR profiles generated from buccal swabs and other sample types such as tissue, blood, and bone (manuscript in preparation) generated using the ANDE System may be utilized for databasing matching or for kinship analysis using local databases. Rapid DNA technology and related legislation and policies have advanced in parallel over the past decade and promise to have a major impact on societal safety.

## Electronic supplementary material

Supplementary Table 1 (XLSX 17 kb)

Supplementary Table 2 (XLSX 10 kb)

Supplementary Table 3 (XLSX 16 kb)
